# Effects of Chinese Formula Jueyin Granules on Psoriasis in an Animal Model

**DOI:** 10.1155/2014/512562

**Published:** 2014-08-04

**Authors:** Tian Ma, Wen-cheng Jiang, Xin Li, Jie Chen, Tie-jun Wu, Hua Nian, Rong Xu, Qian-yuan Huang, Qing-qing Xiao, Qiang Jian, Fu-lun Li, Bin Li

**Affiliations:** ^1^Department of Dermatology, Yueyang Hospital of Integrated Traditional Chinese and Western Medicine, Shanghai University of Traditional Chinese Medicine, 110 Ganhe Road, Shanghai 200437, China; ^2^Department of Pharmacy, Yueyang Hospital of Integrated Traditional Chinese and Western Medicine, Shanghai University of Traditional Chinese Medicine, Shanghai 200437, China

## Abstract

Although Traditional Chinese medicine (TCM) is known to be effective for psoriasis patients, the responsible mechanisms still remain poorly understood. In this study, we aimed to evaluate the effect of one formula, named Jueyin granules (JYG) in the mouse model of the vaginal epithelium and tail epidermis. Additionally, we also determined the anti-inflammatory effects of JYG in an imiquimod- (IMQ-) induced psoriasis-like skin mouse model. Our results show that JYG can attenuate the IMQ-induced psoriasis-like inflammation, accompanied with increased epidermal hyperplasia. We also measured estrogenic stage mitosis of vaginal epithelial cells and the formation of granular cell layers in male mouse tails per 100 scales, as well as the tissue nitric oxide (NO) and malondialdehyde (MDA) levels using the ELISA method. The results suggest that JYG significantly inhibited mitosis in mouse vaginal epithelial cells, promoted the formation of the squamous epidermal granular layer in mice tails, and reduced the levels of NO and MDA in an imiquimod-induced psoriasis-like skin mouse model after 14 d (*P* < 0.05). These results demonstrate that JYG might be an effective clinical treatment for psoriasis and the effects may be related to inhibited keratinocytes proliferation, improved parakeratotic epidermal cells, and reduced expression of NO and MDA.

## 1. Introduction

Psoriasis is a chronic inflammatory skin disease affecting 0.6% to 4.8% of people worldwide [1]. Clinically, psoriasis patients may present with pustular, guttate, or erythrodermic variants that include itching and painful lesions that negatively affect quality of life. The pathogenesis of psoriasis is not completely understood, and the excessive proliferation of keratinocytes in psoriasis patients is an extremely serious health issue. In China, patients with psoriasis often turn to alternative and complementary treatments, which are considered to be effective and safe [2]. Traditional medicine provides front-line pharmacotherapy for many millions of people worldwide, although its use is often viewed with skepticism by the western medicine establishment [3]. The use of herbal medicines to prevent the development and recurrence of psoriasis is widely accepted [4]. JYG, an effective formula founded in the 1950s by Han Xia (a well-known Chinese surgeon), has been used clinically for over 50 years. Though the positive outcomes associated with JYG can be observed clinically, the underlying mechanism for those outcomes is not well understood. Recent studies have suggested that nitric oxide (NO) and malondialdehyde (MDA) may play a part in the pathogenesis of various skin diseases, including psoriasis [5, 6]. Specifically, studies have shown that increased NO and MDA contribute to psoriasis activity [5]. Our previous work has demonstrated that Th1 cells may exert a predominant role in the pathogenesis of different psoriasis syndromes [7]. To better understand the function of JYG, we designed an imiquimod-induced psoriasis model for mice treated with JYG. Investigating the mechanisms underlying the effects of JYG on psoriasis patients will provide laboratory data that can identify potential clinical treatments for psoriasis patients.

## 2. Materials and Methods

### 2.1. Animals and Reagents

Pathogen-free C57BL/6 (8 to 10 wk old) mice of a clean grade were purchased from Shanghai SLAC Laboratory Animal Limited Liability Company. All animals were housed and treated in accordance with institutional guidelines, and the protocols were approved by the Animal Ethics Committee of the Traditional University of Shanghai TCM. Stilboestrol was purchased from the Dalian Meilun Biotech Company, Ltd., and Colchicine was purchased from the Shanghai Sinopharm Chemical Reagent Co., Ltd. White Vaseline was purchased from the Yanwei Medical Chemical Factory. NO and MDA ELISA kits were purchased from the Shanghai Pusheng Biotech Company, Ltd.

### 2.2. Drug Preparation

**Figure 1:**
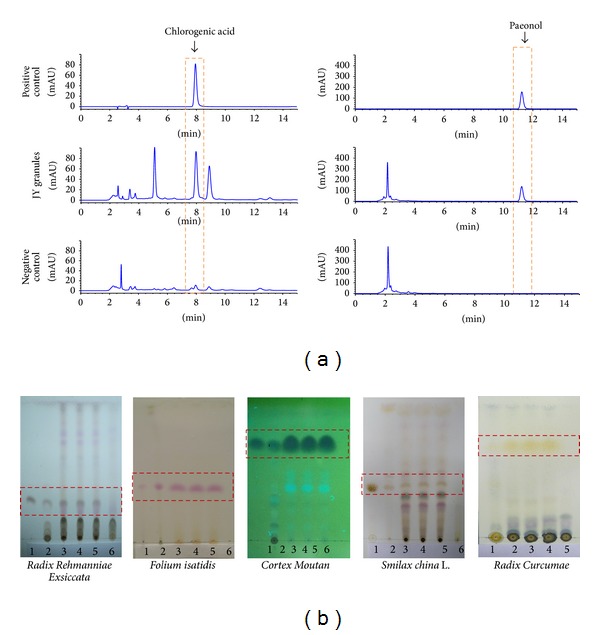
The expression levels of chlorogenic acid and paeonol were used as quality controls of JYG using HPLC. As shown in Figure 1, chlorogenic acid and paeonol were detected in positive control and JY samples, but no expression was found in the negative control (a). Herbs and 3 different batches of JYG products were quantified using thin-layer chromatography. ((b) (1) positive control, (2) herb, (3), (4), and (5) tests of JYG, and (6) negative control). Quality control of JYG by HPLC method.

JYG is comprised of 8 Chinese herbs, as shown in Table 1. The dosage used in the present study was determined according to the Chinese Pharmacopoeia (2010 edition). Granules were prepared by the Jiangsu Tianyin Pharmaceutical Co., Ltd., which adhered to conventional TCM granule methods (water extract-alcohol extraction method). Briefly, (1)* Haliotis diversicolor* was boiled in 500 mL water for 20 min, and (2)* Cortex Moutan* was boiled to collect paeonol crystals using steam distillation. (3) The other 6 herbs were combined and boiled at the highest heat level for another 20 min. (4) The liquid was then transferred using filtration, and another 500 mL of water was added. After bringing the mixture to boil again, (5) the two doses were combined and condensed to a liquid containing 0.6 g~1 g crude drug per milliliter. The water extract was purified using centrifugation at 10,000 revolutions per minute for 15 minutes. (6) The purified concentration was then dried into dry meal by spray drying at a 150°C inlet air temperature and 30% current velocity. (7) The dry meal was then mixed with 19.5% dextrin, 0.5% magnesium stearate, and paeonol crystallization, and JYG was molded using dry granulation at 33 r/min of horizontal-rolled velocity, 5 r/min of round screw die velocity, and 15 MPa of round screw die pressure. Quality control was performed using high-performance liquid chromatography (HPLC) by detecting chlorogenic acid and paeonol. As shown in Figure 1, JYG was demonstrated to have steady quality. JYG was administered at a dose of 0.73 mL/kg/d, which was approximately 7 times the standard dose used in practice (according to the dose-equivalence equation between rats and humans). Compound Indigo capsules produced by the ShanXi Tianning Pharmaceutical Corporation, Ltd., were used as a positive control.

### 2.3. Animal Experiments

As described previously [8], mice between 8 and 11 wks of age received a daily topical dose of 62.5 mg of commercially available 5% imiquimod cream (Sichuan Mingxing Pharmaceutical Corporation, Ltd.) on a shaved back for 6 consecutive days; mice received 3.125 mg of the active compound daily. Forty-eight mice were divided into the following six groups: the control group, the model group, the Compound Indigo capsule (0.68 g/kg/d) treatment group, and the low (0.9 g/kg/d), medium (1.4 g/kg/d), and high (1.8 g/kg/d) dose JYG groups. All mice were treated for 14 days. At the end of the experiment period, the animals were sacrificed and their shaved skins were removed. A rectangular piece of skin situated on the dorsum, 2 cm from the base of the tail, was fixed in paraformaldehyde and processed for subsequent histological examination.

In determining the vaginal epithelium mitosis indexes, sixty mice were divided into groups as above. Stilboestrol was administered by intraperitoneal injection (IP) at a volume of 10 mg/kg/d for 3 days and mice were then treated as described above for 14 days (except in the case of the normal control group). At the 14th day, all mice were scarified by cervical dislocation after injection of colchicine ampules. Vaginas were removed and fixed in paraformaldehyde and processed for subsequent histological examination.

### 2.4. Scoring Severity of Skin Inflammation

Skin inflammation severity was scored using the clinical Psoriasis Area and Severity Index (PASI). Erythema, scaling, and thickening were scored independently on a scale between 0 and 4 as follows: 0, none; 1, slight; 2, moderate; 3, marked; and 4, very marked. The cumulative score of erythema, scaling, and thickening was used to measure inflammation severity (scales 0–12) [8].

### 2.5. Pathology

Skin samples were fixed in 10% formalin for 24 h at 4°C and then placed in 70% ethanol. All samples were embedded in paraffin, cut into 4 *μ*m sections, and stained with hematoxylin and eosin (H&E).

### 2.6. Effect on the Formation of Granular Layer in Mouse Tail Scale Epidermis

Five sequential cells in the granular layer were considered one scale. The tail scale epidermis was measured from the dermoepidermal junction to the base of the horny layer. The amount of the mouse tail scale epidermis was then calculated to give the number of scales containing a granular layer per 100 scales in each mouse.

### 2.7. Effects on the Mitotic Index of Mouse Vaginal Epithelium

An ocular micrometer was used to count the number of mitotic cells per length of mucosa as measured along 100 basal layer vaginal cells. The mitotic index was calculated as the arithmetic mean of measurements taken from 100 basal layer vaginal cells.

### 2.8. Assay of Tissue NO and MDA Levels

Skin lesions were weighed, washed three times with normal saline, and homogenized in four volumes of ice-cold buffer (20 mM Tris and 10 mM EDTA (pH 7.4)). The washed and buffered tissue samples were immediately stored at −80°C for later MDA and NO measurement. MDA and NO were measured using commercially available ELISA kits according to the manufacturer's instructions (Shanghai Pusheng Biological Technology Co., Ltd.). Briefly, total nitrite (NO_*x*_) was quantified by the Griess reaction after incubation of supernatant with* Escherichia coli* nitrated reductase to convert NO_3_ to NO_2_. The Griess reagent was then added to 1 mL of supernatant and the absorbance read at 545 nm; this value was compared with standard NaNO_2_ obtained by reducing NaNO_3_. The reaction was linear from 0.25 to 100 *μ*mol/L. To complete the MDA assays, 0.5 mL tissue homogenate was added to a reaction mixture (1.0 mL) composed of equal parts of 15% trichloroacetic acid, 0.25 N HCL, and 0.375% thiobarbituric acid (TBA), plus 2.5 mM butylated hydroxytoluene (BHT) and 0.1 mL of 8.1% sodium dodecyl sulphate (SDS). The mixture was heated for 30 min at 95°C; the pH value of the analytical reaction mixture was approximately 0.9. After cooling, chromogen was extracted using N-butanol and compared spectrophotometrically at 532 nm with a blank reaction mixture, which lacked tissue homogenization supernatants but was subjected to the entire procedure and extracted with N-butanol. The results were expressed as nmol/g in wet tissue according to a standard prepared using serial dilutions of standard 1,1,3,3-tetramethoxypropane (see Figure 5).

### 2.9. Statistical Analysis

All data are presented as mean and standard deviation (mean ± SD); significant differences were analyzed using the unpaired, 2-tailed Student's *t*-test. Pearson's correlation coefficient was used to determine the correlation between PASI score and NO and MDA levels. A *P* value less than 0.05 was considered to be significant.

## 3. Results

### 3.1. Effect of Jueyin Granules on Imiquimod-Induced Psoriasis Model

**Figure 2:**
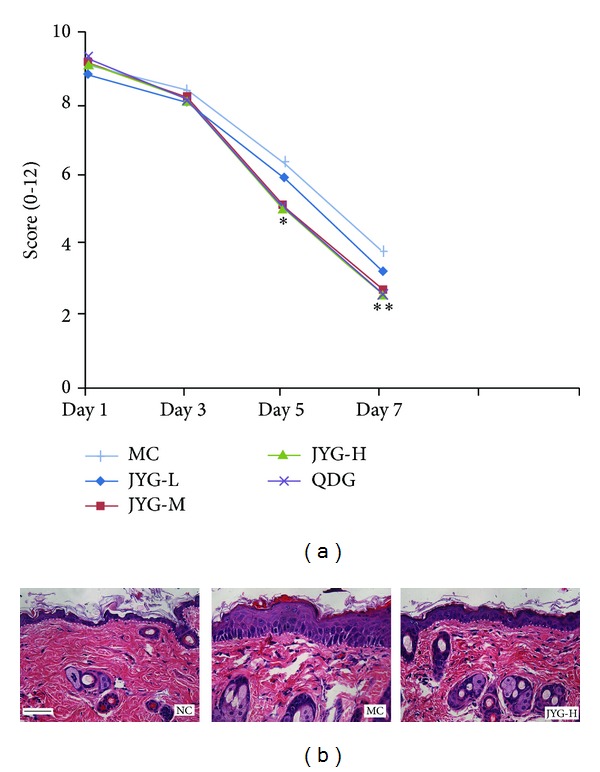
The effect of JYG on IMQ-induced skin inflammation in mice. (a) Cumulative inflammation score (erythema plus scaling plus thickness) on day 7 is recorded. **P < 0.05 compared with MC; *P < 0.05 compared with JYG-L. (b) H&E staining of skin lesions. IMQ treatment alters keratinocyte proliferation and differentiation, while JYG relieved those changes. Dotted lines delineate the epithelial-stromal boundary. The scale bar in the first panel represents 100 *μ*m for all sections.

To observe the anti-inflammation effects of JYG, mice with imiquimod-induced psoriasis were used. IMQ cream was applied on shaved back skin for 6 consecutive days. As a result, the mice in the normal control group lived actively and showed quick reactions. Their hair was shiny and the skin had no erythema or scaling. Three days after IQM application, the back skin of treated mice started to display signs of erythema, scaling, and thickening. After 6 days, the inflammation intensified. Both the JYG and Qingdai capsule-treated groups showed fewer symptoms of inflammation and reduced scores, as shown in Figure 1(a). However, the JYG-H and -M groups, but not the JYG-L group, exhibited anti-inflammatory activity. JYG suppressed IMQ-induced psoriasis (as measured by PASI score) in a dose-dependent manner. The inflammation scores are shown in Figure 1(b). Compared with the model group (8.33 ± 0.71), inflammation scores were significantly decreased in the other groups. The QDG and JYG-L, -M, and -H experiment groups had inflammation scores of 5.78 ± 0.67, 5.44 ± 0.73, 6.67 ± 0.5, and 6.89 ± 0.6, respectively; there was a significant difference between the scores of the high dosage JYG group and the low dosage JYG group. A similar inhibitory effect was observed with respect to epidermal thickness. Further analysis using H&E staining indicated that IMQ-treated skin showed increased epidermal thickening caused by hyperproliferation of keratinocytes. In the high dosage JYG group, acanthosis was significantly reduced, as shown in Figure 2(b).

### 3.2. Effect of Jueyin Granules on the Formation of Granular Layers in Mouse Tail Scale Epidermis

**Figure 3:**
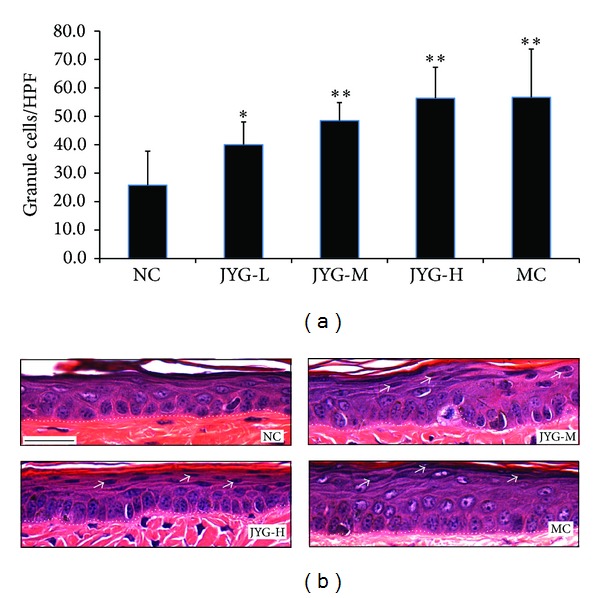
The effect of JYG on the stratum granulosum in mice tails. (a) Mice were treated with JYG at a concentration of 0.901 g/kg, 1.352 g/kg, or 1.802 g/kg for 14 days (*n* = 6). The number of stratum granulosum lodicules was then measured. NC: normal control; MC: model control; JYG-L: JYG low dosage; JYG-M: JYG middle dosage; JYG-H: JYG high dosage. (b) Photos of H&E staining of mice treated with different doses of JYG. Dotted lines delineate the epithelial-stromal boundary. *P < 0.05 and **P < 0.01 compared with NC. The scale bar in the first panel represents 100 *μ*m for all sections.

Compared with the value in the negative control group (25.759 ± 11.98), the values of the QDG, JYG-L, -M, and -H groups were 56.7 ± 16.9, 56.4 ± 10.9, 48.50 ± 6.3, and 40.03 ± 8.02, respectively (*P* > 0.05). In the negative control group, a small number of particles were found in shallow epidermal cells. However, in the JYG treated groups, a larger number of particles were arranged in a layer of cells within the tail shallow epidermis, and the formation of granular cell layers in mice tail scales was significantly promoted. No significant granular cell layer difference was found between the positive control group and the high dosage JYG group. The results are shown in Figure 3.

### 3.3. Effect of Jueyin Granules on Mitosis of Vaginal Epithelium at Estrum

**Figure 4:**
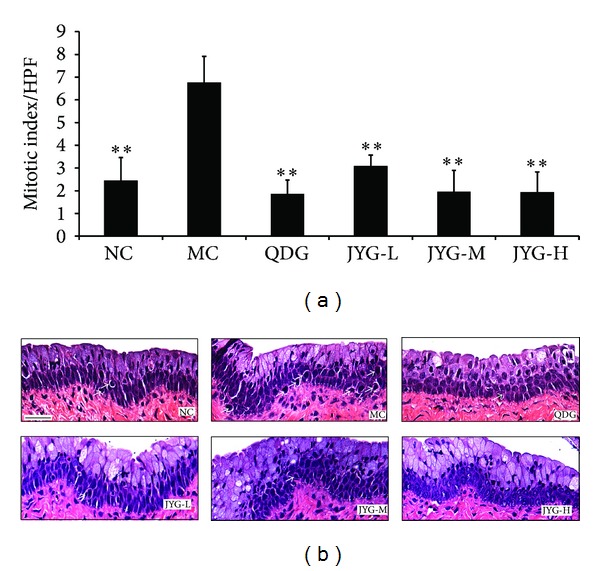
The effect of JY granule on the mitosis of vagina epithelium cells in mice (a) and preventive pictures. (b) Histograms represent mitosis scores for each group treated with a different JY formula, *n* = 6. (b) H&E staining of skin lesions from each group. Dotted lines delineate the epithelial-stromal boundary. **P < 0.01 compared with MC. The scale bar in the first panel represents 100 *μ*m for all sections.

**Figure 5:**
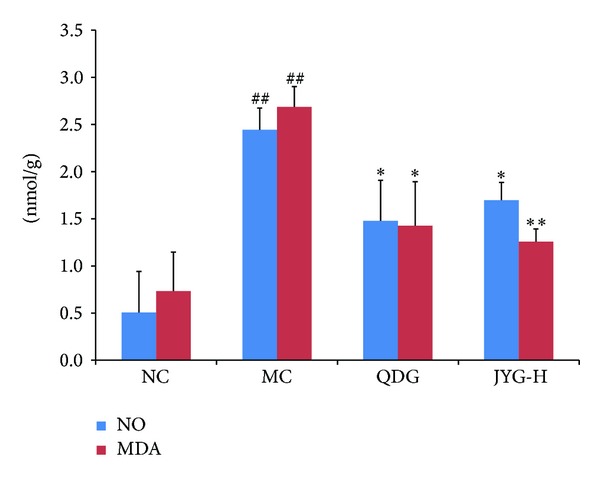
The mean levels of NO and MDA expression in tissue from different experiment groups. MDA and NO in homogenized tissue were measured using ELISA kits. *P < 0.05; **compared with MC; ^##^ *P* < 0.01 compared with NC.

In the negative control group, many mitotic cells were observed (2.46 ± 1.01), with cellular nuclei swelled, condensed, and heavily dyed. Compared with the negative control group, there were significantly fewer mitotic cells in the other treated groups. As shown in Figure 4, the mitotic index of the negative group was significantly higher than each experiment group as well as the positive control group (6.77 ± 1.15, 3.087 ± 0.49, 1.96 ± 0.94, 1.93 ± 0.90, and 1.87 ± 0.60, resp.), while no significant mitotic index differences were found between the JYG-M group, the JYG-H group, and the positive control group.

### 3.4. Influence of Jueyin Granules on the Expression of NO and MDA in Mice with Imiquimod-Induced Psoriasis

Levels of NO and MDA (2.44 ± 0.23 and 2.69 ± 0.22 *μ*mol/L, resp.) were significantly higher in psoriasis lesion tissue than tissue from the normal control group (0.51 ± 0.43 and 0.73 ± 0.41 *μ*mol/L, resp.). JYG treatment reduced levels of NO and MDA in inflammatory lesions to 1.69 ± 0.19 and 1.26 ± 0.14 *μ*mol/L, respectively (*P* < 0.05); there were no significant differences between the JYG treatment group and the positive drug control group (1.48 ± 0.43 and 1.42 ± 0.47 *μ*mol/L, resp.; *P* > 0.05). A significant correlation was found between PASI score and levels of both NO and MDA. After 4 weeks, the expression levels in the model group continued to approach those of the normal group (data not shown).

## 4. Discussion

Psoriasis lesions are characterized by accelerated epidermal proliferation and the infiltration of inflammatory cells into the superficial dermis. Moreover, inflammatory changes in the skin frequently lead to reduced barrier function resulting from aberrant epidermal cell differentiation. Several hypotheses regarding psoriasis have been proposed by researchers, who have suggested that activated T lymphocytes, tumor necrosis factor *α* (TNF-*α*), IL17/IL23, and so forth play a crucial role in psoriatic inflammatory changes. However, the exact pathogenesis of psoriasis remains unclear. In 1971, Bonder and van Scott [9] first reported that mouse vaginal and rectal epithelia provide systems by which antimitotic properties of drug candidates can be examined. This model is widely used in psoriasis research [10, 11]. Modern medicine has made breakthrough progress in psoriasis treatment, with glucocorticoid, immunosuppressive, retinoic acid, and biological preparations all currently available. However, those traditional systemic therapies have a well-documented array of toxicities which limit their clinical application. Hence, there has been a demand for new therapeutic methods.

Cortisone like betamethasone is a hormone, which has stronger anti-inflammatory effect than Chinese herbs. Conversely, the side effect is much more severe than that of herbs. Abuse of corticosteroids aggravates psoriasis and is well-known to dermatologist. The treatment of psoriasis is still a controversial issue. For many years, clinical doctors have suggested that TCM formulas are effective and safe in the treatment of psoriasis [1, 4, 12]. Patients with psoriasis have usually been divided into three groups according to the TCM differentiation of blood treatment theory: blood heat, blood stasis, and deficiency of blood. Treatment with TCM is less costly compared with narrow-band UVB, psoralen plus UVA, retinoid, alefacept, and other new biologic agents [13].

In China, doctors often prescribe a combination of plant species called formulae, based on TCM theories, to enhance therapeutic efficacy and reduce adverse effects. JYG, containing 8 different herbs, is a well-known formula created in the 1950s by Han Xia (a well-known Chinese surgeon). Our previous clinical research has shown that this formula has a clinical effect of approximately 83.3% on blood-heat type psoriasis and improved patient's life quality [14]. Additionally, our data also suggest that downregulation of NO and MDA is largely responsible for JYG-mediated inflammation relief. Based on the TCM theory and our experience, we believed the pathogenesis of this type of psoriasis patient had the characteristics of long time internal overheating or suffering from fire and heat evil, or lack of Yin due to mistreatment. Then, evil heat would be forced into the blood and blocked in the skin. Our clinical experience indicates that* Flos Lonicerae japonicae* and* Haliotis diversicolor* are the principal components of the formula, while* Radix Rehmanniae exsiccata*,* Cortex Moutan*, and Herba* Hedyotis diffusae* serve as minister drugs.* Folium Isatidis *and* Smilax china *L. were used as adjuvant ingredients, while* Radix Curcumae* acts as envoy drug. In order to further explore its efficacy in treating inflammation and discover the possible underlying mechanism for its action, we used three different animal models to provide detailed laboratory data on JYG. Granule quality was controlled under high-performance liquid chromatography (HPLC). As the results show, 2-week treatment with the JYG and Qingdai capsules significantly improved scaling, erythema, and induration compared with the negative control. Furthermore, the effects were dose dependent. The sum of scaling, erythema, and induration scores in the lesion area of the JYG group was 18% lower than the control group.

Imiquimod is an effective ligand for TLR7 and also interferes with adenosine receptor signaling [15]. It has been used for the topical treatment of genital and perianal warts caused by human papilloma virus [16]. However, it can exacerbate psoriasis in well-controlled patients [17]. Investigators have confirmed that IMQ-induced skin inflammation is associated with the IL-23/IL-17A axis and can serve as a model for analyzing the pathogenic mechanisms associated with psoriasis-like dermatitis [8]. This effect on mice skin closely resembles human psoriasis and presents the erythema, scaling, and epidermal alteration observed in our study. Our results indicate that JYG inhibited the increase in skin thickness and inflammation in IMQ-treated mouse skin, which is caused by hyperplastic basal suprabasal keratinocyte.

Skin is a major target of oxidative stress due to ROS originating from the environment and skin metabolism [17]. Serum NO and MDA levels have previously been reported to be significantly elevated in psoriasis patients [5]. Yildirim reported that no statistically significant difference was found between the serum MDA levels of psoriasis patients and those of healthy controls [17]. However, an increase in reactive oxygen species (ROS) and insufficient antioxidant activity have been shown in psoriatic lesions. In our study, NO and MDA were elevated in imiquimod-induced inflamed skin, and both JYG and QDG reduced the expression levels.

In summary, we demonstrated that JYG is an effective remedy for treating psoriasis in a mouse model, and these effects were the result of inhibited keratinocyte proliferation and reduced NO and MDA expression. Because of the complexity of YJG formula, its role on multiple targets is not clear. Further investigation is necessary to identify the main functional composition, clarify the molecular mechanisms responsible for the multiple effects of JYG, such as antiproliferation and inflammatory factor expression, and search for new efficiency element to dampen the inflammation process.

## Figures and Tables

**Table 1:** Ingredients of JYG used with English translations.

Medicine and dose used	English translation
*Haliotis diversicolor *	Concha Haliotidis
15 g	

*Flos Lonicerae * * japonicae *	Honeysuckle flower
12 g	

*Radix Rehmanniae exsiccata *	Dried *Rehmannia* root
15 g	

*Cortex Moutan *	Tree peony bark
12 g	

Herba *Hedyotisdiffusae *	*Oldenlandia *
15 g	

*Folium isatidis *	Dyer's woad leaf
15 g	

*Smilax china *L.	Chinaroot greenbrier rhizome
15 g	

*Radix Curcumae *	Turmeric root tuber
9 g	
